# OBITUARIES

**Published:** 2010

**Dr. Pritam Singh**

Dr. Pritam Singh, ex-president and editor ISA breathed his last at Cincinnati USA on 16^th^ October 2009. Apart from being the President of the ISA in 1970 and the Editor of IJA from 1964 to 1970, he held various prestigious posts like visiting Professor of the University of Cincinnati College of Medicine, Ohio, USA; Professor and HOD of Anaesthesiology at CMC, Ludhiana; Principal of Govt. Medical College, Amritsar and Director of Research and Medical Education of Punjab. He had served the Indian Army Medical Corps from 1942 - 1946 and rose to the rank of Captain

**Figure:**
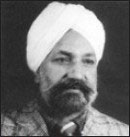
20^th^ June 1920–16^th^ Oct 2009

**Dr. G Subramanian**

With profound grief, Dindigul City Branch of the TN Chapter ISA reports the sad demise of Dr. G Subramanian on 29^th^ November 2009 at the age of 70 years. An ISA life member, Dr G. Subramanian was the founder President of Dindigul ISA, TN state. He is survived by his wife and a son.

**Figure:**
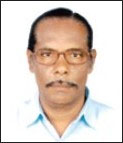
10^th^ Dec 1940 – 29^th^ Nov 2009

